# A Deformational Analysis of a Titanium Alloy Supported by the Mathematical Modelling of the Sheet Metal Forming Process via Numerical Simulation

**DOI:** 10.3390/ma18071598

**Published:** 2025-04-01

**Authors:** David Koreček, Pavel Solfronk, Jiří Sobotka

**Affiliations:** Department of Engineering Technology, Faculty of Mechanical Engineering, Technical University of Liberec, Studentská 1402/2, 46117 Liberec, Czech Republic; pavel.solfronk@tul.cz (P.S.); jiri.sobotka@tul.cz (J.S.)

**Keywords:** light alloys, mechanical properties, titanium, yield criterion, hardening model, spring-back, sheet metal forming, FEM, numerical simulation, PAM-STAMP 2G

## Abstract

The issue of the mathematical modelling of the deformation process nowadays affects an indispensable part of the industrial stamping production process. In view of this issue, this paper focuses on the research and analysis of the mechanical properties and stress–strain characteristics of the titanium alloy AMS4900. The investigated material properties and characteristics serve as input parameters defining the yield criterion in the environment of sheet metal forming process numerical simulations. Through the quality of the input parameters and the choice of the material model, the accuracy of the resulting numerical simulations is quite fundamentally affected. For this reason, the influence of the selected material models and the relevant material parameters with respect to the real sheet metal forming process is compared and evaluated in this paper.

## 1. Introduction

The ecology, economics, rate and productivity of production are the trends that drive the world’s industrial production today, including the automotive industry. These general requirements and assumptions are forcing car manufacturers to increasingly address the issue of introducing new types of materials for the production of more than just car body stampings. There is a tendency to use materials with a favourable strength/ductility ratio, which contributes to ensuring the low specific weight of the produced parts. The low weight of these parts contributes directly to reducing the final weight of the car and thus helps to reduce the production of CO_2_ and other greenhouse gases. At the same time, this trend is placing ever-greater demands on the complexity and shape of the stampings, which is directly related to the perfect mapping of the production process before the production begins. A correct description of the deformation and, above all, the subsequent spring-back of the stamping parts is key to ensure an economical and productive production process that is able to react flexibly to current issues, problems and possible process changes during production. To address these issues, software based on a numerical FEM simulation of the sheet metal forming process is now widely used. In order to ensure the correct computations of the numerical simulation, it is very important to have correct and accurate settings of the technical process itself, which must correspond to reality. In addition to this, there is also a need to have the most accurate material data, which involves the choice of the material computational model and, last but not least, the setting of boundary conditions and other process parameters.

The issue of numerical simulations is closely related to the choice of a suitable material computational model that will be able to describe the deformation process and the subsequent material spring-back after the unloading of the pressing tool with sufficient accuracy. In order to achieve accurate results of a numerical simulation correlated with the course of the real forming process, it is necessary to first select a suitable type of yield criterion within the computational model, which defines the transition of the material from an elastic to plastic state, and then define a suitable material hardening model in the area of plastic deformation. These and other issues related to numerical simulations and the mathematical modelling of manufacturing processes are currently being addressed by various research institutes all over the world. The investigated issues can be found, for example, in the publications of Prof. Henk Vegter from Tata Steel Europe Limited [1,2,3,4,5,6,7], Prof. Pavel Hora from ETH Zurich [8,9,10,11,12,13], Prof. Takeshi Ue-mori from Okayama University [14,15,16,17,18,19], Assoc. Takeshi Yoshida of Hiroshima University [19,20,21,22,23,24,25] and others.

One of the very important aspects and problems in the mathematical modelling of the material deformation behaviour in the elastic, elastic–plastic and plastic region is undoubtedly the consideration and description of their anisotropic behaviour [26]. The anisotropy of a material influences the position of the so-called boundary of the yield criterion or the boundary of the material transition from an elastic to plastic state during its deformation. For a correct description of the material anisotropic behaviour, it is necessary to take into account the different directions and states of stress in the material being formed with respect to the given forming process. These studies are currently being carried out, for example, by Prof. Henk Vegter and others [5,7,10,27]. These and other studies show the advantage of using advanced yield criteria in numerical simulations of the forming process, which take into account the influence of anisotropy and different loadings and stress states in the material during deformation.

An indispensable issue related to the yield criterion is further represented by the material hardening model during its deformation [26]. During the forming process, and especially during the sheet metal forming process, with deformation and different directions of loading, the material changes yield strenght, where the tensile stress changes to compressive stress and vice versa. For this reason, in this case, the utilization of the isotropic hardening model is relatively incorrect and inaccurate. The application of the so-called Bauschinger effect, which describes the change in the yield strength of the material during alternating cyclic tension/compression loading, is involved. This phenomenon complicates to a large extent the mathematical formulation of the material hardening model in the region of plastic deformation and is also quite closely related to the defined yield criterion. In the case of the requirement to achieve accurate deformation and spring-back results in numerical simulations, it appears to be advantageous to use a model that can describe and define the non-isotropic hardening model during its deformation [26]. To describe the change in the yield stress under alternating loading and to define the influence of this change on the hardening process, models involving the kinematic hardening of the material have been developed and proposed. This is described, for example, in Yoshida-Uemori [28]. In the experimental research carried out, as described by Yoshida [28], the observation of cyclic plasticity has only been made in a limited number of publications, as this requirement and its main development only arose with the current boom in numerical simulations of the sheet metal forming process [28]. Another decelerating element of this issue is surely the considerable complexity of performing cyclic material testing, used to obtain the stress–strain characteristics of the material under alternating tensile–compressive loading and the subsequent description and definition of the kinematic hardening model [28]. The design and testing of the cyclic loading methodology is investigated, e.g., in the publication by Prof. Yoshida [29]. Specifically, the deformation behaviour of high-strength material during cyclic loading is investigated and the design of a correct methodology for this method of material testing is presented. This method of loading the material can be seen as a change in the strain path together with the Bauschinger effect, representing the effect associated with a change in yield strength, when the material is loaded in different directions [26]. In general, the assumption that can be considered is that the deformation at the beginning of the process does not only affect the position and magnitude of the yield stress but also its shape [26]. This topic is further discussed and investigated in publications such as those of Barlat et al. (2013) [30], Feigenbaum et al. (2012) [31] and Freund et al. (2012) [32]. This issue was also investigated in [33] but only for simplified Vegter Lite material models and for a significantly different material type in the form of steel.

The research presented in this paper aims to build on the previously mentioned studies of this issue and to link the area of the mathematical modelling of stamping and subsequent spring-back with the possibility of using and developing such a methodology for advanced types of materials such as titanium alloys. The forming of titanium alloys is not yet a fully and clearly investigated area and the results of this research can certainly contribute to the research development about the mechanical behaviour of these materials and further developments in the mathematical modelling of sheet metal forming in the case of special material types. So far, titanium alloys have been investigated from the perspective of forming, for example, in publications by Yanxi Li [34], Camilo A.F. Salvador [35], Y. Danard [36], Ramin Hosseini [37] and C. Brozek [38], where the deformation and deformation mechanisms of titanium alloys are investigated. In the publication of M.C. Zang [39] and Zhiying Liu [40], the mechanical properties of alpha alloys are also evaluated using the static tensile test. However, the connection between the titanium alloys mechanical testing results and the subsequent implementation of these data for the mathematic modelling of forming processes has not yet been fully elucidated. The major contribution of this study is certainly in the application of experimentally obtained data to the environment of the sheet metal forming process numerical simulations, where (acc. to the Vegter yield criterion) the deformation computation is based on experimental data and not on the theoretical mathematical general assumptions.

Another contribution is certainly the application of the so-called Yoshida kinematic hardening model, which allows us to verify the calculation of the forming process in light of the kinematic hardening law in the area of plastic deformation based on the implementation of results from performed cyclic tests and thus contribute to numerical simulation result improvements, e.g., see the above-mentioned publications of prof. Yoshida [28] and [29]. Compared to the previously mentioned publications, the present study has the advantage of showing and allowing the application of these complex yield criteria for special material types (in this case, a titanium alloy), which, these days, have quite a lot of applications. On the other hand, the possibilities of their applications in industrial practice are so far limited due to many problems accompanying the forming process such as lubrication and galling problems [41] and especially the problematic deformability and extreme spring-back of titanium and its alloys. This study can certainly be helpful in many areas and support the existing research in the field of metal forming titanium and its alloys and further support the possibilities of their application in industrial practice.

## 2. Materials and Methods

**Figure 1:**
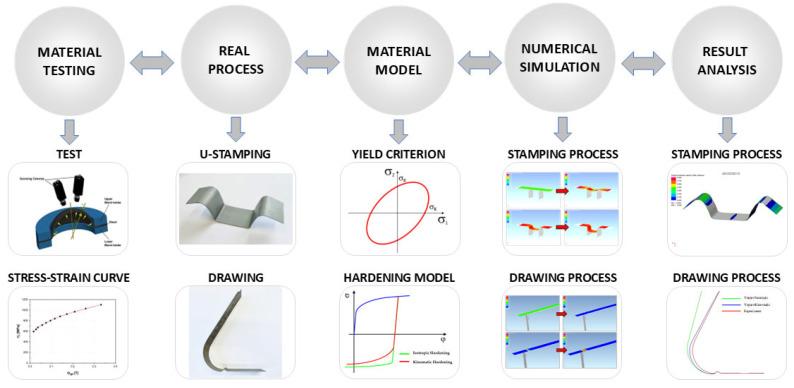
A graphical abstract of the individual steps of this research.

The research submitted in this study is based on the linking material analysis and numerical modelling of the production process (in this case, stamping) and the subsequent material spring-back. The whole experimental layout and the individual steps of our research are shown in Figure 1.

### 2.1. Material

#### Titanium Grade 3 AMS4900

The element titanium was first discovered in 1971. It was discovered by the British chemist William Gregor, and originally, titanium was named Gregorite after him. Independently from this discovery, this element was discovered two years later by the German chemist M.H. Klaproth, who named it titanium after the Titans of Greek mythology [42,43]. The process of successfully isolating titanium was not known until 1910 [42]. The development of processing titanium and its alloys only began to play an important role from about the middle of the 20th century, when it was used to meet the requirements of aircraft engine designs [44] and for other military applications. The first practical use of titanium dates back to 1948 in the USA, where 2 tonnes of this material were produced for the first time [44]. Titanium production further started in the former USSR in 1950 [44].

Nowadays, titanium and its alloys are mainly used for their high strength, low specific weight and corrosion resistance. At the same time, the use of titanium has mainly found its place in the aerospace industry and other special applications [43,44,45]. However, there are still growing demands from automotive manufacturers for the use of titanium and its alloys in the automotive sector, where the aforementioned properties of titanium make it possible to reduce, e.g., vehicle weight, improve the durability and functionality of certain parts, reduce fuel consumption, etc., which also leads to improved environmental performance and a reduction in the carbon footprint of the car [41,46,47].

The titanium element forms two basic allotropic modifications. The first structure is characterized by a hexagonal crystal lattice. This structure is stable up to a temperature of 882.5 °C. The second allotropic modification is characterized by a body-centred cubic crystal lattice and is stable from 882.5 °C to a melting temperature of 1668 ± 4 °C [44,48,49,50]. The α phase of titanium is the primary phase, and the temperature of its allotropic transformation to the β phase is largely influenced by the purity of titanium. While for iodide titanium, the temperature is at the mentioned boundary of 882.5 °C, for commercially pure titanium, the allotropic transformation takes place in the temperature range of 860 to 960 °C [51,52]. At low heating and cooling rates, the phase transformation proceeds by nucleation and the subsequent growth of new crystals, while at high heating and cooling rates, the allotropic modification has features of a martensitic transformation [51]. Low cooling rates lead to the formation of a cellular microstructure due to the disordered, diffusive growth of the new phase, and then at higher cooling rates, a needle-like microstructure is formed [51].

Grade 3 titanium (AMS4900) belongs to α titanium alloys, and it is essentially pure titanium, referred to as CP (commercial pure). Titanium α alloys are characterized by their good strength, fracture toughness and rupture resistance, even at very low temperatures, and they also possess high creep resistance at high temperatures and retain sufficient strength when exposed to temperatures up to about 300 °C [44,48,53,54].

### 2.2. Mechanical Testing of Material

This chapter describes selected material tests of AMS4900 titanium. These tests are used to load the tested material under specific stress states, defining the plasticity yield criterion and hardening law of the material during plastic deformation. The results of the material testing serve as very important input data for the definition of material models in the numerical simulation environment of PAM-STAMP 2G 2022.0 software.

#### 2.2.1. Static Tensile Test

**Figure 2:**
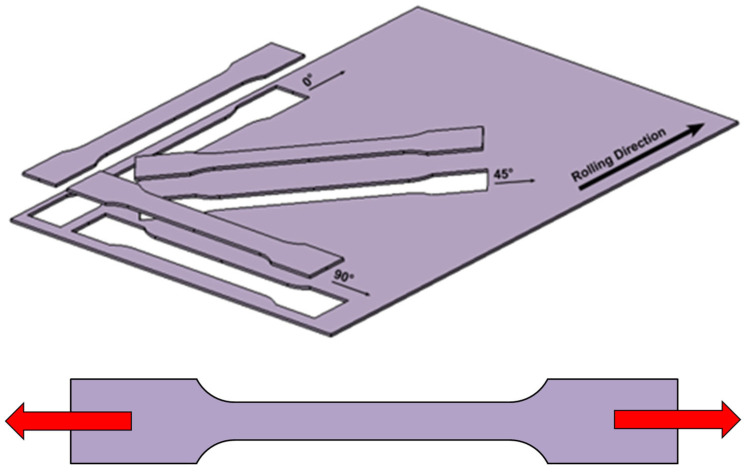
Graphical illustration of selected directions and loading scheme for static tensile test.

The static tensile test is one of the basic material tests simulating the uniaxial state of stress tension, namely uniaxial tension. The results of this test are used to define the uniaxial stress in the plane of principal stresses σ_1_ and σ_2_. The test was performed for specimens taken in the 0°, 45° and 90° directions relative to the rolling direction of the tested sheet. The test was carried out according to standard EN ISO 6892-1. An illustration of the specimens used for the selected directions and their loading during the static tensile test is shown in Figure 2. The testing specimens were made with initial dimensions as follows: *L*_0_ = 80 mm, *b*_0_ = 20 mm and *t*_0_ = 0.6 mm. The test was carried out at a loading rate of 1 mm/min, common ambient temperature of 23 °C and relative air humidity of 50%.

The static tensile test was carried out on a device, TiraTest 2300 (Schalkau, Germany). The force was determined by a head force sensor, KAF 20 kN from the company Angewandte System Technik GmbH (Aschheim, Germany), and the absolute elongation was recorded by the integrated axial extensometer MFX-500-B. The specimen was clamped by the hydraulic jaws and loaded by a continuously increasing axial force. The test was carried out up to the moment of its failure, which expressed the absolute elongation at the moment of failure, or more specifically, the total elongation *A*_80mm_. The data recording and evaluation of the measured quantities were performed in LabNET 4.49.8945 and ORIGIN 2020 software.

#### 2.2.2. Hydraulic Bulge Test

**Figure 3:**
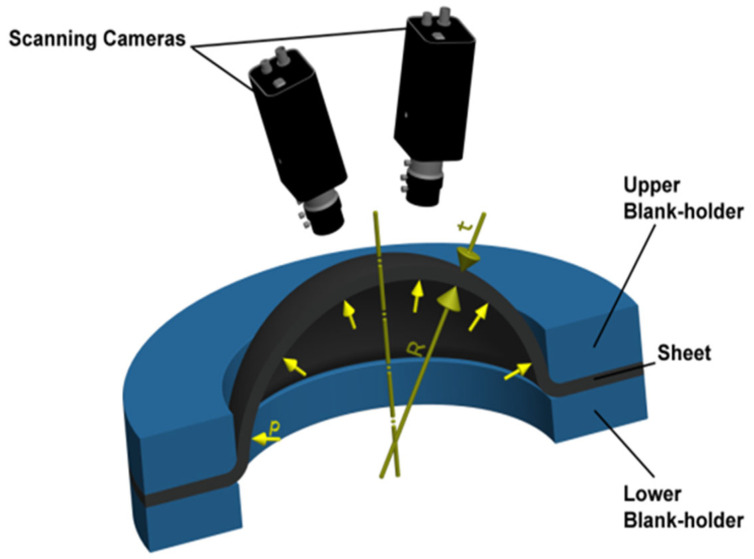
Schematic illustration of hydraulic bulge test.

The so-called hydraulic bulge test is a test where the tested material is loaded by equi-biaxial stress induced by the pressure of the used hydraulic medium. The results of this test are used to define the biaxial stress in the plane of the principal stresses σ_1_ and σ_2_. This test was carried out for rounded specimens (diameter = 210 mm) cut out from the tested sheet. A schematic of this test is illustrated in Figure 3. The test was carried out at a loading rate of 1 mm/min, common ambient temperature of 23 °C and relative air humidity of 50%.

The test was carried out on a CBA300/63 hydraulic press (TOS Rakovník, Rakovník, Czech Republic) using a jig, enabling the proper performance of this test. In this case, the blank is loaded by the force of hydraulic pressure, clamped between the upper and lower blank holder. Subsequently, the specimen is loaded by hydraulic pressure, which ensures the equi-biaxial stretching of the blank up to the moment of rupture. During the test, the pressure was scanned by a pressure sensor placed in the jig, and strain values were recorded by a pair of stereo cameras using an optical system MERCURY RT (from the company SOBRIETY, Maidstone, UK). So, these strain values were detected by a photogrammetric method, where a random greyscale pattern was applied on the surface of the specimen. During the test, this pattern was scanned by the cameras and optical system, where the imaged area was divided into so-called “facets” and the proper position of these facets was identified during deformation based on the assigned greyscale value.

#### 2.2.3. Plane Strain Tensile Test

**Figure 4:**
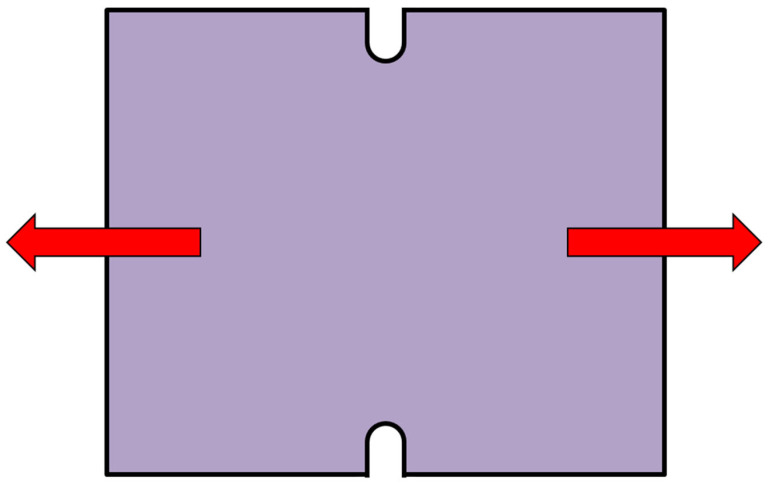
A schema of the plane strain tensile test.

A special plane test is used to simulate a stress state, where the specimen is subjected to a load while the strain in the width direction equals zero (*φ*_2_ = 0). This condition is ensured by the geometry of the specimen—see Figure 4. The results of this test are used to define the stresses *σ*_ps_ in the plane of principal stresses σ_1_ and σ_2_. This test was also performed for the specimens in the selected directions 0°, 45°and 90° with respect to the rolling direction of the tested material. The test was carried out at a loading rate of 1 mm/min, common ambient temperature of 23 °C and relative air humidity of 50%.

The test was carried out also using the TiraTest 2300 static tensile testing machine. The force was sensed by a KAF 100 kN head force sensor from Angewandte System Technik GmbH, and the deformation was measured and evaluated with a contactless optical system from the SOBRIETY company. The specimen was clamped during the test by the mechanical jaws and loaded by a continuously increasing axial force applied by the testing machine. The test was terminated at the moment of specimen fracture or the appearance of a crack in the notch area.

#### 2.2.4. Shear Test

**Figure 5:**
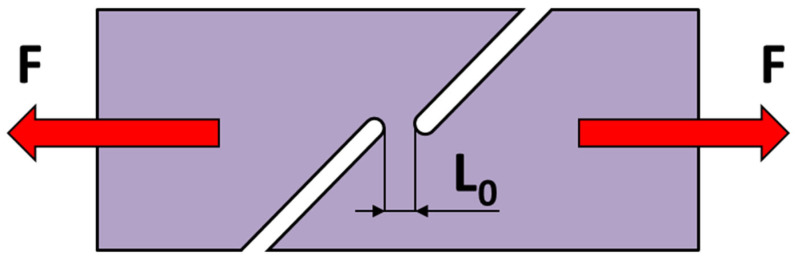
A schema of the shear test.

The shear test (or plane shear test) is used here when the testing specimen is subjected to shear stress. This load, due to the geometry of the specimen (see Figure 5), occurs here in the form of a simple plane shear state of stress right in one shear plane (American Standard ASTM B831). The test body is loaded during the test by a continuously increasing force, induced by the translational movement of the testing device grips. The test is carried out until a crack appears right in the shear plane.

Again, testing specimens were prepared in the directions 0°, 45° and 90° with respect to the rolling direction. The test was also carried out using a TiraTest 2300 static tensile testing machine—similarly to the plane strain test (see above). Here, the force was sensed using a KAF 20 kN head force sensor from Angewandte System Technik GmbH, and the deformation was also measured and evaluated by the optical system SOBRIETY. The test was carried out at a loading rate of 1 mm/min, common ambient temperature of 23 °C and relative air humidity of 50%.

#### 2.2.5. Cyclic Test

**Figure 6:**
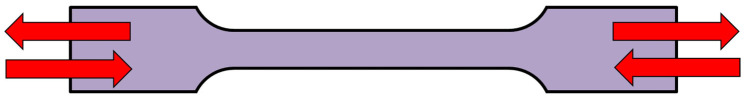
A schema of the cyclic test.

As a final test to determine the material properties using a specific loading method, a cyclic alternating load test was used. This test is used to define the material properties under alternating tension and compression cyclic loading and is also used to define a model of material hardening in the region of plastic deformation, where the influence of the Bauschinger effect, which is manifested by the change in the yield stress during the transition from tensile to compressive stress in the deformed material, is taken into account. The loading of the material is shown schematically in Figure 6. Our test was performed under the following conditions: a loading rate of 10 mm/min, an amplitude of ±3 mm and three and five cycles. The test was carried out at a loading rate of 1 mm/min, common ambient temperature of 23 °C and relative air humidity of 50%.

**Figure 7:**
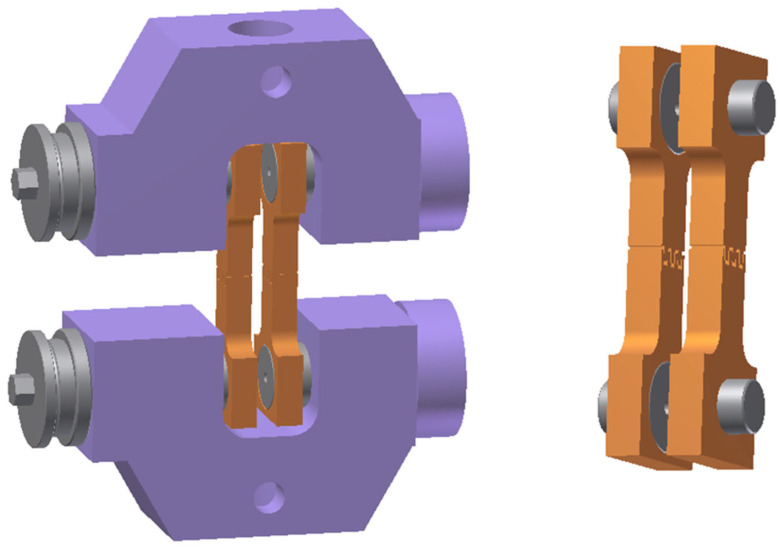
Special grips for the cyclic test.

For this test, standard testing specimens, as used in the static tensile testing, were used. The test was carried out in a similar way to the static tensile test using the TIRA Test 2300 testing machine. During the test, the specimen was clamped using a special fixture to prevent its buckling due to the loading during compression (see Figure 7).

During the test, the force was sensed by a KAF 20 kN head force sensor from Ange-wandte System Technik GmbH as in the static tensile test, and the elongation/contraction of the specimen was measured by the axial extensometer MFX-500-B. The test was carried out for a given number of working cycles, alternating between tensile and compressive loading. The data recording and subsequent evaluation of the measured quantities were performed in LabNET and ORIGIN 2020 software.

### 2.3. The Fabrication of the Real Stampings

As the last part of the experimental research, real stampings were made to serve as a comparative specimen (“etalons”) and thus to verify the results from the numerical simulation of the sheet metal forming process. Real stampings were prepared for two simple cases of sheet metal forming. Using simple forming tools, we produced stampings corresponding to the U-shaped bending process and stampings produced by the sheet being drawn over the draw-bead.

#### 2.3.1. U-Shaped Drawing

**Figure 8:**
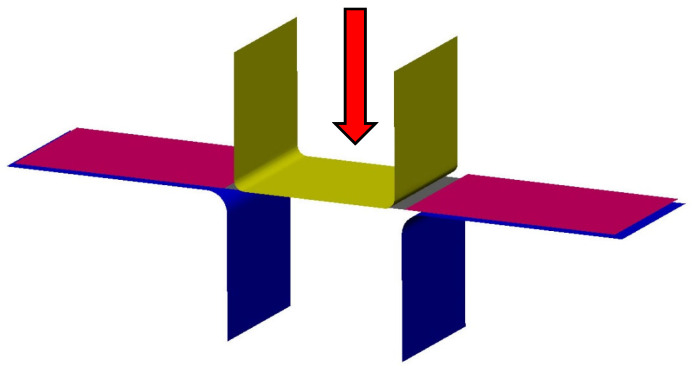
A schema of the U-shaped bending.

A simple metal forming tool consisting of a drawing punch, drawing die and a blank- holder was used to draw the blank in the U shape, as shown in Figure 8. This experiment was chosen to represent a truly simple forming process, where the testing material underwent a relatively small deformation through a single deforming zone in the drawing die radius region. Our bending test was carried out for a stroke of 30 mm at a loading rate of 5 mm/min.

**Figure 9:**
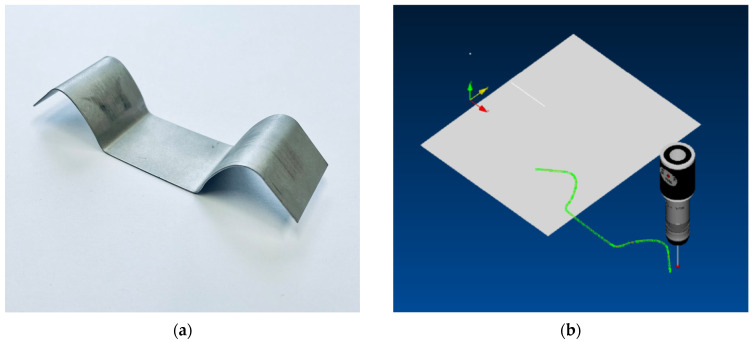
Illustration of U-shaped stamping after spring-back (**a**) and contour scanning of this stamping (**b**).

The real stampings—see Figure 9a—had to be transferred into the form of a CAD model for their comparison with the numerical simulation results. The contour of the stamping was always scanned using a SOMET XYZ 464 3D coordinate measuring device and TANGO!3D 2.0 software (see Figure 9b).

**Figure 10:**
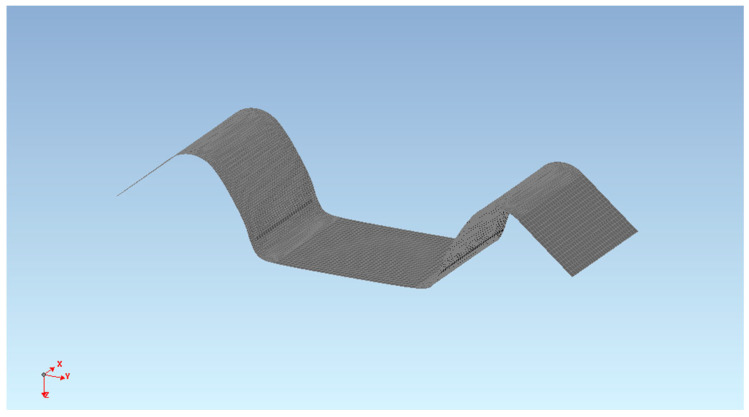
An illustration of the digital form of a real U-shaped stamping.

The detected points on the real stamping were subsequently smoothed with a curve in CATIA V5 software, and a mathematical surface of the real stamping was created from this curve, which was then imported into the PAM STAMP 2G numerical simulation software for comparison purposes (see Figure 10).

#### 2.3.2. Drawing over the Draw-Bead

**Figure 11:**
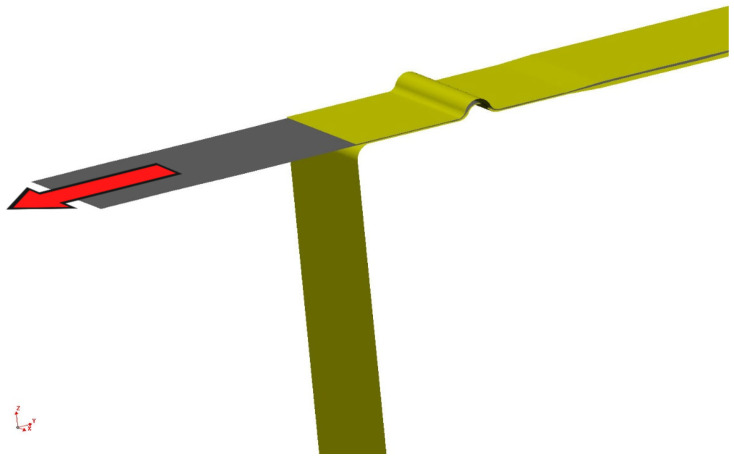
A diagram of the strip drawing test with the application of the draw-bead.

Furthermore, a second experiment was carried out, where a sheet testing strip was drawn over a draw-bead on the tribological testing machine Sokol 400 (Technical University of Liberec, Liberec, Czech Republic). The sheet was drawn over the draw-bead by means of an axial sliding motion of the testing tool hydraulic jaw. The draw-bead was formed by means of interchangeable shape inserts. A schematic representation of the process is illustrated in Figure 11.

**Figure 12:**
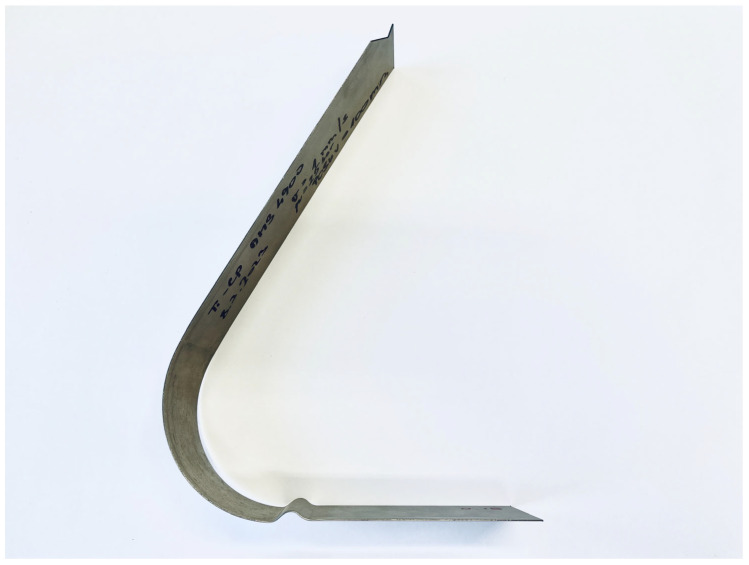
An illustration of the real stamping after its drawing over the draw-bead.

This type of experiment is chosen because, here, the material undergoes a relatively large deformation with a repeated change in the stress state in the testing specimen. The draw-bead in this drawing process ensures a change in tensile and compressive loads during the passage of the testing material, i.e., the Bauschinger effect is applied here as in the common deep drawing of sheet metal stampings. Because of this, a significant influence of the chosen material hardening law in the area of plastic deformations can be expected. After fabrication, the contour of the stamping was again scanned using a 3D measuring device, and then a digital twin was made, which again was used to make a comparison with the results of the numerical simulation. The real stamping after the subsequent spring-back of the material is shown in Figure 12.

### 2.4. Numerical Simulation

#### 2.4.1. The Finite Element Method (FEM) in the Sheet Metal Forming Process

Most of the modern software applications that deal with the numerical simulation of forming processes are based on computations of the finite element method. This method is based on the principles of non-rigid body mechanics and is one of the most accurate tools for the computation of metal forming numerical simulations. In contrast to classical variational methods, the FEM is based on an approximation of the quantity being investigated. The resulting function is constructed only in limited regions (volumes) by means of non-zero approximations. These regions are created by dividing them into geometrically simple disjunctive regions: elements. Regions in the plane are usually converted into triangles or polygonal elements. Volume regions are then converted into tetrahedra, cubes, etc. The basis of these elements is called the finite element mesh. Given the number of elements contained in this basis, it is possible to find just enough approximations to model the function using the “spline” curves. The functions used for approximation are generally chosen to be as simple as possible, often constituting, e.g., polynomial dependence, where the number of arguments depends on the type of problem. Outside of the individual elements, the functions are defined as zero, and the continuity requirement of the function must be satisfied at the element boundaries. This is conditioned by the dependence of the combined coefficients of the elementary functions for the individual prescriptions of the approximation of adjacent elements. This sub-problem is taken into account via the elimination of the individual coefficients and their functional values in properly chosen regions of the elements, referred to as nodes, which are preferably located at the boundaries or vertices of the chosen elements [55,56].

The practical computation of the forming process in the numerical simulation is carried out by converting all components involved in the computation into a computational mesh of the given elements. For a component involved in the computation but not subject to the deformation process, the mesh represents only the geometry of this component. Conversely, for deformable objects involved in the deformation process, these elements have well-defined behaviour under the influence of a load and other process-related parameters. The mechanical phenomena related to deformation are generated in each individual element and thus give a comprehensive result for the behaviour of the whole object. An element can exist in the form of a two-node element (bar), a three-node element (triangle), a four-node element (quadrangle), or an element of six or eight nodes. Each node has two types of degrees of freedom—translation and rotation. As the number of elements increases, the computational accuracy of the numerical simulation increases, but on the other hand, the hardware complexity and individual computation times increase rapidly [57,58].

Depending on the type of simulation (implicit or explicit), the numerical computation is divided into increments or time steps. At the node points of the elementary “finite element” mesh, kinematic quantities such as position, velocity and acceleration as well as load-related quantities such as force are detected at each moment. These values are further converted at each node into actual stress and strain values. For a realistic representation of the component deformation, the simulation must be supplemented with material characteristics and dimensions of the deformed component [57,58].

#### 2.4.2. Computational Material Models in PAM STAMP 2G Software

Material computational models in the numerical simulation environment generally express and contain information about the behaviour of a given material under its loading. These include, in particular, the definition of the basic physical material characteristics and the definition of the stress–strain characteristics of the given material. The definition of the stress–strain behaviour of the material during the forming process is based on the chosen yield criterion, which controls the transition of the material from the elastic to the plastic state, and on the hardening model in the area of plastic deformation. A suitable definition of the yield criterion in numerical simulations can be taken into account in several ways, and isotropic or anisotropic conditions can be defined. Furthermore, it is possible to use conditions where the computation of the material deformation behaviour is solved by a mathematical approach (e.g., Hill, Barlat, etc.) or by externally specified (“fitted”) material characteristics, which are provided by means of material testing (e.g., the Vegter model, Yoshida, etc.) [58].

**Figure 13:**
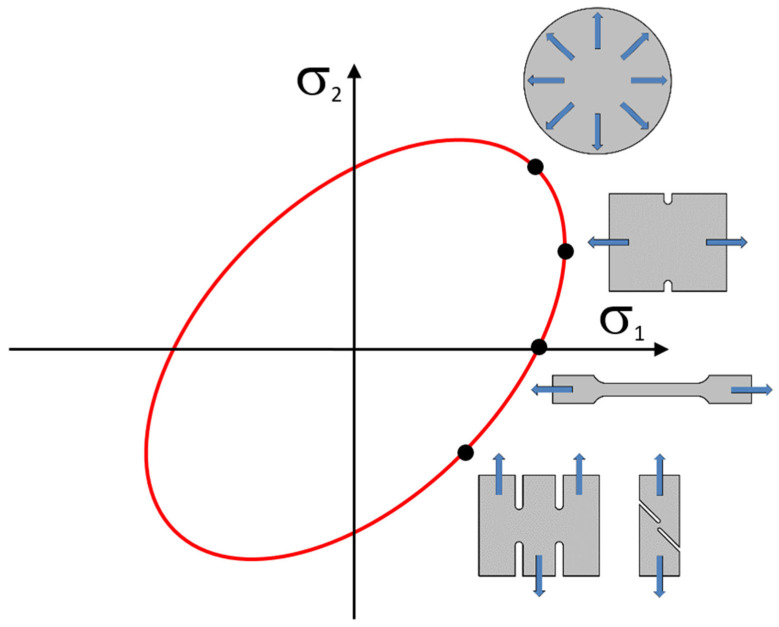
Hinge (control) points of the ellipse represented by different material tests.

In the case of computing the forming of thin sheets, stress σ_3_ is not considered during the computation and the yield conditions thus takes a planar form, where it is expressed by an ellipse, which forms the yield criterion boundary within the plane of the main stresses σ_1_ and σ_2_. The yield criterion boundary, and thus the shape of this ellipse, is controlled by mathematical expressions solving the anisotropy of the material or by the hinge points obtained by selected material tests (see Figure 13) [58].

In the case of a simple yield criterion, this boundary condition is represented only by hinge points determined by the static tensile testing, which corresponds only to the material yield strength (e.g., the Hill 48 model) and Lankford’s coefficient [58]. Other parameters for determining this boundary are solved by using mathematical formulations. The Hill 48 model is one of the simplest yield criteria and, in conjunction with the isotropic hardening law of the material, is very easy to be defined. Its mathematical formulation is based on Hill’s Equation (1) [59,60], where *F*, *G*, *H*, *L*, *M* and *N* are material constants regarding the state of material anisotropy and x and y are axes of anisotropy [59,60]. Since only the plane state of stress is considered in the numerical simulations of the sheet metal forming processes, this formula can be simplified (see Equation (2)), where *σ*_1_ and *σ*_2_ are principal stresses and *r*_0_, *r*_45_ and *r*_90_ are Lankford’s coefficients [60]. This model is included in this study, taking into account that it is the simplest one to make comparisons with the advanced Vegter model.

(1) $$2f(\sigma_{ij})={F(\sigma_{y}-\sigma_{z})}^{2}+{G(\sigma_{z}-\sigma_{x})}^{2}+{H(\sigma_{x}-\sigma_{y})}^{2}+2L\tau_{yz}^{2}+2M\tau_{zx}^{2}+2N\tau_{xy}^{2}=1,$$

(2) $$\sigma_{1}^{2}-\frac{2.r_{0}}{1+r_{0}\;}{\;\sigma }_{1}\;\sigma_{2}+\frac{r_{0}\;(1+r_{90})}{r_{90}\;(1+r_{0})\;}\sigma_{2}^{2}=\sigma_{0}^{2}$$

For the definition of more complex conditions and a more accurate description of the material transition to the plastic state, it is possible to extend the material tests by, for example, bi-axial tests, special planar tests or shear tests to more accurately describe the different regions of the yield criterion boundary condition, just by using more hinge points (see Figure 2). This can be carried out, e.g., via the Vegter model, whose plasticity boundary is then defined by the points obtained from the material testing (see Figure 2). These individual points are here defined as the ratio of the stress obtained via a specific loading method of the material to a reference stress obtained from the static tensile test *σ*_un_ (0°). The resulting envelope of the plasticity boundary is then formed by linear interpolation.

The selected material models used in the numerical simulations depend on two main material states. The first is the transition of the material from an elastic to a plastic state; this is defined by the chosen yield criterion. The subsequent material behaviour in the plastic deformation region is described by a material hardening model due to its deformation. Both of these factors significantly influence the overall accuracy and the validity of the numerical simulation results of the forming process.

**Figure 14:**
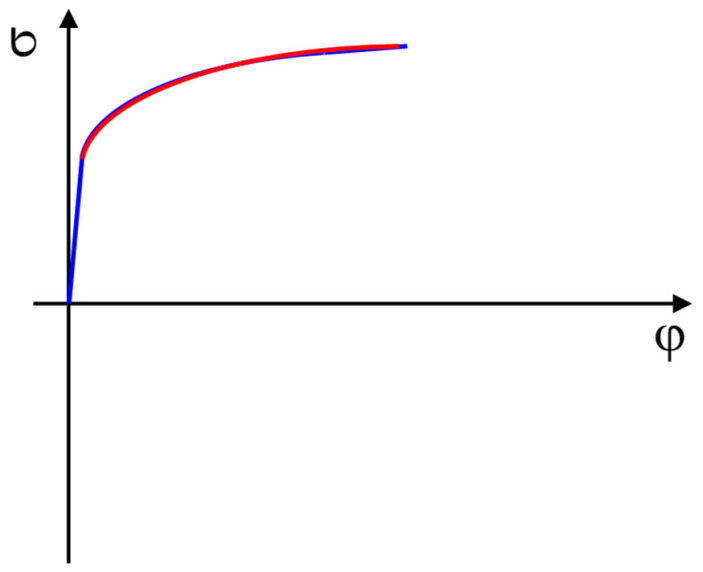
An approximation of the stress–strain curve measured by the static tensile test.

The material hardening due to its deformation can be described in several ways. The simplest model is the so-called isotropic hardening model. This model works on the basis of approximating the stress–strain curve determined by the static tensile test (see Figure 14). The approximation is made by fitting the measured curve with the curve defined by the Krupkowski equation—see Equation (3).

(3) $$\sigma ={C\;\cdot \;\left(\varphi +\varphi_{0}\right)}^{n}$$

where

*C*—strength coefficient (MPa);
*n*—strain hardening exponent (-);
*φ*_0_—offset true strain (-).

**Figure 15:**
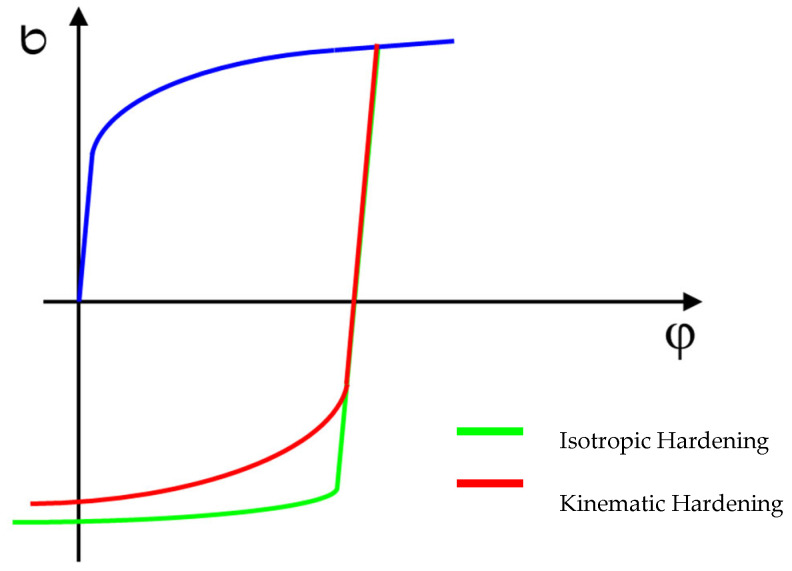
Kinematic vs. isotropic hardening model during material deformation.

For a more accurate description of the material hardening model in the area of plastic deformation, the kinematic hardening model (the so-called Yoshida model) is used. This model no longer considers isotropic hardening law but is based on a change in yield stress in the case of the transition from tensile to compressive stress and vice versa, i.e., it takes into account the so-called Bauschinger effect. The application of this phenomenon can be seen in the real sheet metal forming process, e.g., on the drawing edge or in the draw-bead tool. The definition of this model is carried out by means of a measured curve forming hysteresis loops measured by cyclic testing under a fully reversed (tensile–compression) cyclic loading of the material. An illustration of a comparison between the isotropic and kinematic hardening model during deformation can be seen in Figure 15.

**Figure 16:**
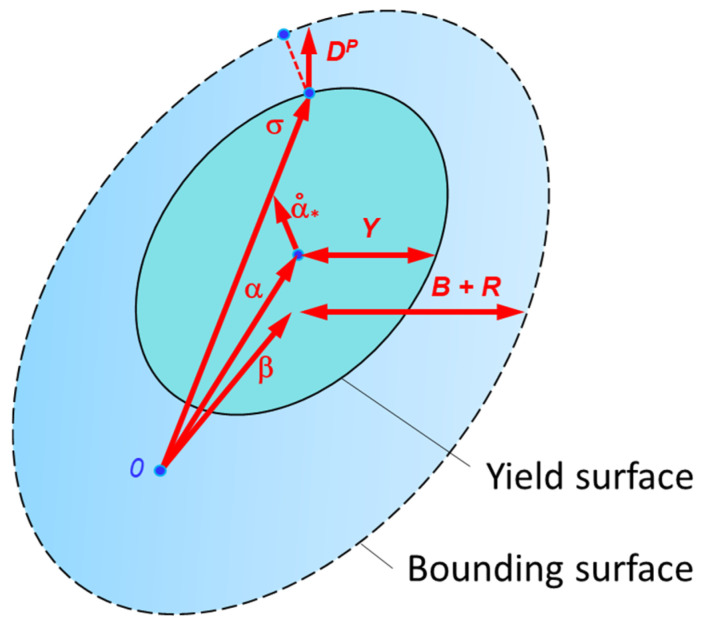
Shift in Yoshida yield surface. $\dot{\alpha }$_*_ is the relative kinematic motion of the yield surface with respect to the bounding surface.

The Bauschinger effect affects not only the material strengthening during deformation but also the yield criterion itself, which defines the bounding surface. This boundary is thus shifted by the Bauschinger effect, as illustrated in Figure 16.

The anisotropic condition of the Vegter yield criterion is then defined via Equations (4)–(6), where σ_1_ and σ_2_ express the principal stresses and angle φ expresses the rotation of the coordinate system in the plane of these principal stresses [58]. This model was included in this research study, taking into account that it serves as a representative of the advance yield criteria, which should more precisely define the material’s transition to the plastic state.

(4) $$\sigma_{1}=\;\;\frac{\sigma_{xx}+\sigma_{yy}}{2}+\sqrt{{\left(\frac{\sigma_{xx}-\sigma_{yy}}{2}\right)}^{2}+{\sigma_{xy}}^{2}}\;$$

(5) $$\;\;\sigma_{2}=\;\;\frac{\sigma_{xx}+\sigma_{yy}}{2}-\sqrt{{\left(\frac{\sigma_{xx}-\sigma_{yy}}{2}\right)}^{2}+{\sigma_{xy}}^{2}}\;$$

(6) $$\cos(2ϴ)=\;\;\frac{\frac{\sigma_{xx}-\sigma_{yy}}{2}}{\sqrt{{\left(\frac{\sigma_{xx}-\sigma_{yy}}{2}\right)}^{2}+{\sigma_{xy}}^{2}}}\;\;$$

where

*σ*_1_—principal stress (direction 1) (MPa);
*σ*_2_—principal stress (direction 2) (MPa);
*σ*_xx_—stress in the direction 0° (MPa);
*σ*_yy_—stress in the direction 90° (MPa);
*σ*_xy_—shear stress (MPa);
*ϴ*—angle of the coordination system rotation (°).

**Figure 17:**
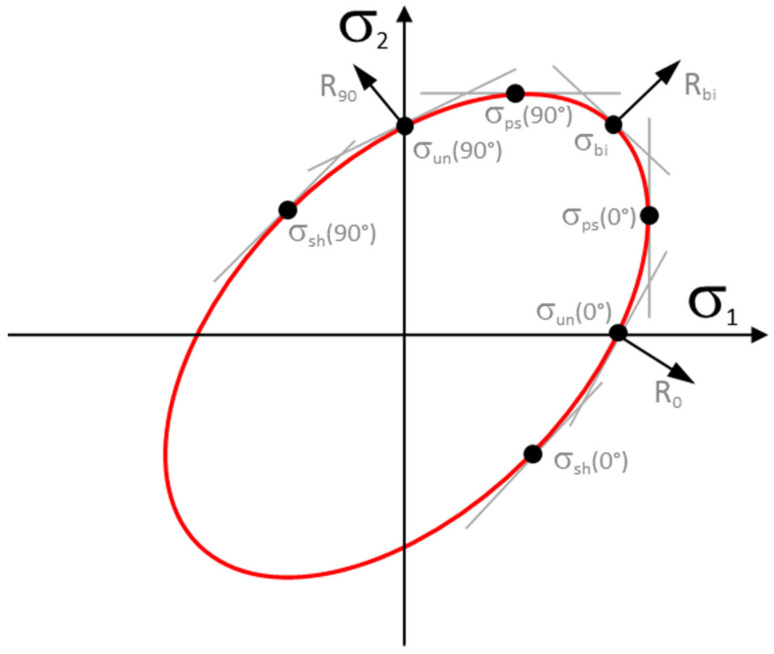
Vegter yield criterion—boundary conditions.

In PAM-STAMP 2G 2022.0 software, the Vegter yield criterion (see Figure 17) can be defined in its full version with the inclusion of all the required material tests. This model is then defined by using the following:

- Young’s modulus *E*;
- Poisson’s ratio *μ*;
- Density *ρ*;
- Normal anisotropy coefficients *r*_0_, *r*_45_ and *r*_90_;
- Uniaxial stress *σ*_un_(0°) and *σ*_un_(90°);
- Bi-axial anisotropy coefficient *r*_bi_;
- Bi-axial stress *σ*_bi_;
- Plain strain–stress *σ*_ps_(0°), *σ*_ps_(45°) and *σ*_ps_(90°);
- Shear strain–stress *σ*_sh_(0°), *σ*_sh_(45°) and *σ*_sh_(90°).

In addition, the material hardening model in the plastic deformation is defined by approximating the stress–strain curve from a static tensile test for the isotropic hardening model or by approximating the stress–strain curve from a cyclic test for the kinematic hardening model (the so-called Yoshida model).

## 3. Results

### 3.1. A Metallographic Evaluation of the Structure of the Tested Material

**Figure 18:**
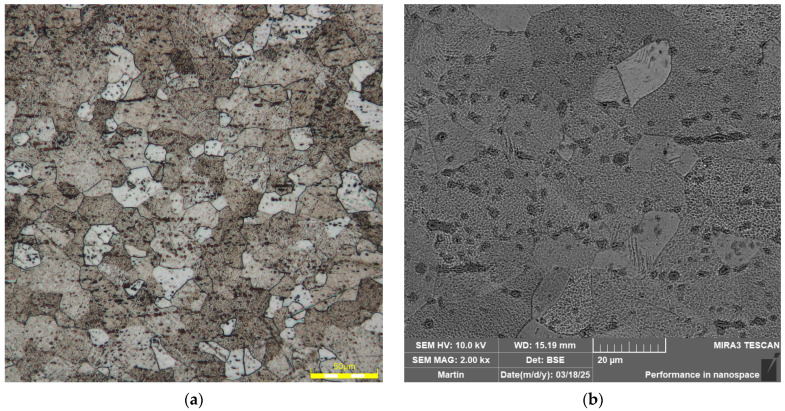
Structure of the tested material shown by optical microscopy (**a**) and structure shown by SEM (**b**).

Figure 18 illustrates the microstructure of the material Ti-CP AMS 4900. The input material is in the form of an annealed sheet. Figure 18a shows the microstructure of the tested material using an Olympus DSX510 optical microscope (Waltham, MA, USA). Then, (b) illustrates the structure using SEM analysis on a Tescan MIRA 3 (Brno, Czech Republic). It can be seen that the grain structure of the tested material is fine-grained with equiaxed grains. From the SEM analysis, it can be seen that titanium-based precipitates with low iron content are present in the material matrix.

### 3.2. Mechanical Tests of Titanium AMS4900

#### 3.2.1. Static Tensile Test

**Figure 19:**
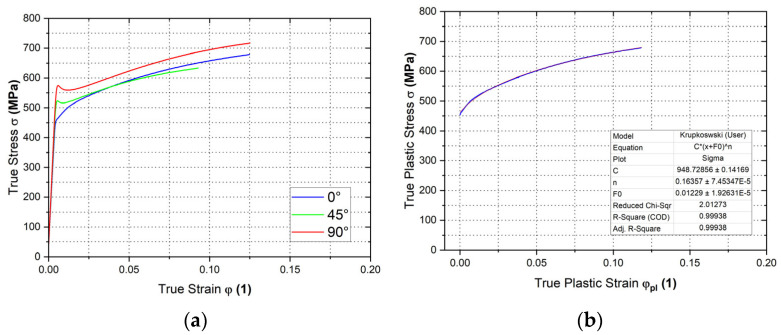
The stress–strain curve of Titanium AMS 4900 from the static tensile test (**a**) and Krupkowski approximation in the rolling direction 0° (**b**).

The graphs in Figure 19 illustrate the resulting dependencies of true stress–strain curves determined by the static tensile test. Moreover, we also applied the Krupkowski approximation to such curves to obtain relevant coefficients (see Equation (3)).

The basic mechanical properties of Titanium AMS4900 obtained from static tensile testing are shown in Table 1. Furthermore, Table 2 shows the approximation coefficients determined by the Krupkowski approximation for the selected directions of 0°, 45° and 90° with respect to the rolling direction.

#### 3.2.2. Hydraulic Bulge Test

**Figure 20:**
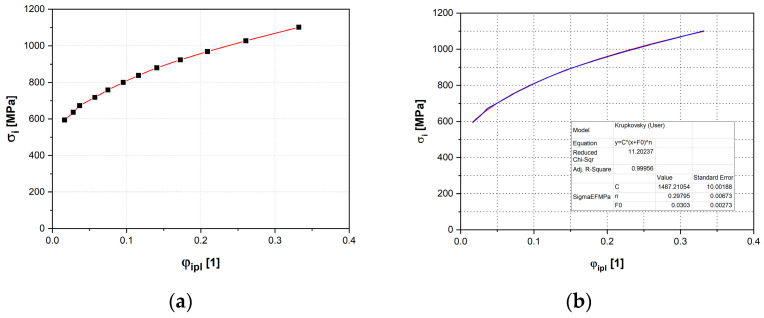
The stress–strain curve of Titanium AMS 4900 from the HBT (**a**) and the Krupkowski approximation (blue colour) of the relevant stress–strain curve (red colour) (**b**).

The graphs in Figure 20 illustrate the resulting dependencies of the effective stress and effective strain obtained from the HBT and the Krupkowski approximation of the hardening curve to determine the approximation coefficients—see Equation (3)—which are shown together with the anisotropy coefficient from this test in Table 3.

#### 3.2.3. Plane Strain Tensile Test

**Figure 21:**
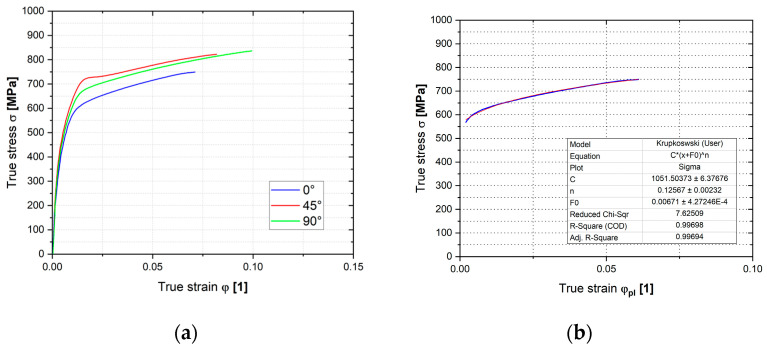
The stress–strain curve of Titanium AMS 4900 from the plane strain tensile test (**a**) and the Krupkowski approximation (blue colour) of the relevant stress–strain curve (red colour) (**b**).

Graphs in Figure 21 illustrate the resulting dependencies of the true stress–strain curves obtained from the plane strain test and the Krupkowski approximation of the hardening curve to determine the approximation coefficients—see Equation (3)—which are shown for each rolling direction in Table 4.

#### 3.2.4. Shear Test

**Figure 22:**
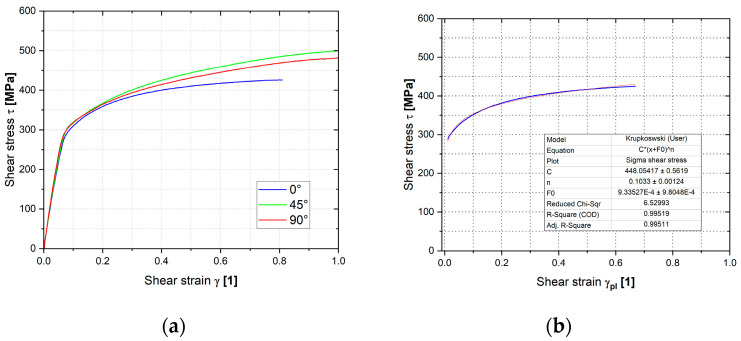
The stress–strain curve of Titanium AMS 4900 from the shear test (**a**) and the Krupkowski approximation of the relevant stress–strain curve (**b**).

The graphs in Figure 22 illustrate the resulting dependencies of the true stress and strain determined by the shear test and Krupkowski approximation of this hardening curve to obtain the approximation coefficients (see Equation (3)), which are shown for each rolling direction in Table 5.

#### 3.2.5. Cyclic Test

**Figure 23:**
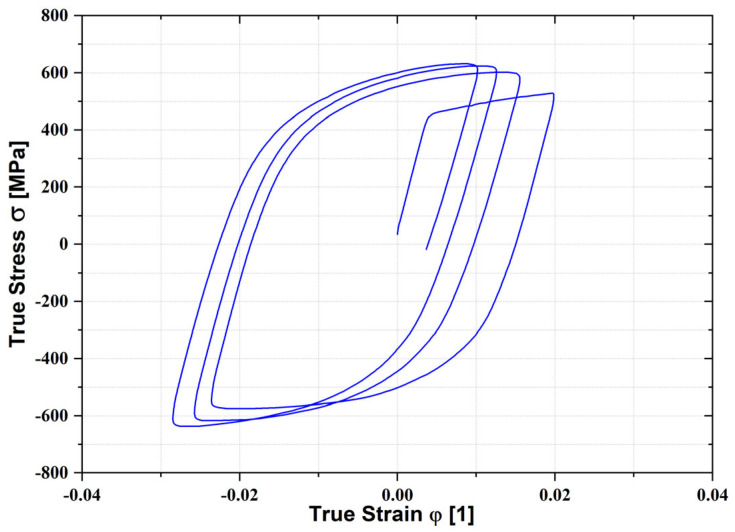
The stress–strain curve of the material ASM4900 from the cyclic test (rolling direction 0°).

The graph in Figure 23 shows the resulting dependence of the true stress–strain curve, which creates a hysteresis loop, which is further used to define the kinematic hardening model, as it is described in the following sections. Differences between the yield strengths regarding the tensile–compression loading changes are shown in Table 6.

### 3.3. Numerical Simulation

#### 3.3.1. Material Model Definition in Software PAM STAMP 2G

The implementation of the determined material data and selected characteristics is carried out in the software PAM STAMP 2G on the basis of so-called “fitting”. As described above, these quantities are input into the environment of the numerical simulations as a material model. Firstly, the basic material properties have to be specified, then the yield criterion needs to be defined, and finally, the hardening law needs to be defined.

**Figure 24:**
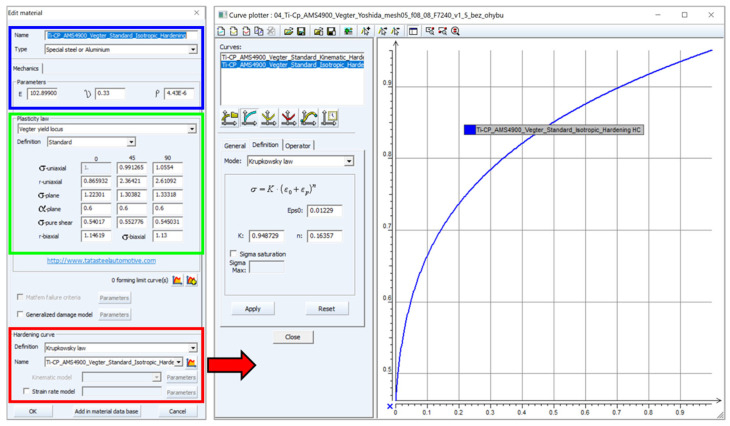
The definition of the yield criterion (the Vegter one) in the PS2G environment (**left**) and definition of the isotropic hardening law (**right**).

Figure 24 below shows the definition of the chosen Vegter material model (the yield criterion). In the blue-marked area, the basic material properties such as Young’s modulus of elasticity E, Poisson number ν and density ρ are specified. In the green-marked area, our yield criterion is defined using the results from the individual material tests. The stress characteristics from the individual material tests (different types of loading—see the mechanical testing—always using an average of five measurements) are entered here as the ratio of this stress to the stress from the static tensile test in the reference 0° direction (the rolling direction). This resulting ratio was obtained by comparing the equivalent deformation energies for each loading method with the reference static tensile test in the 0° direction. In the area marked in red, an isotropic hardening law is defined by means of the coefficients obtained by means of the Krupkowski approximation of the stress–strain curve, determined by the static tensile test and again in the 0° direction.

**Figure 25:**
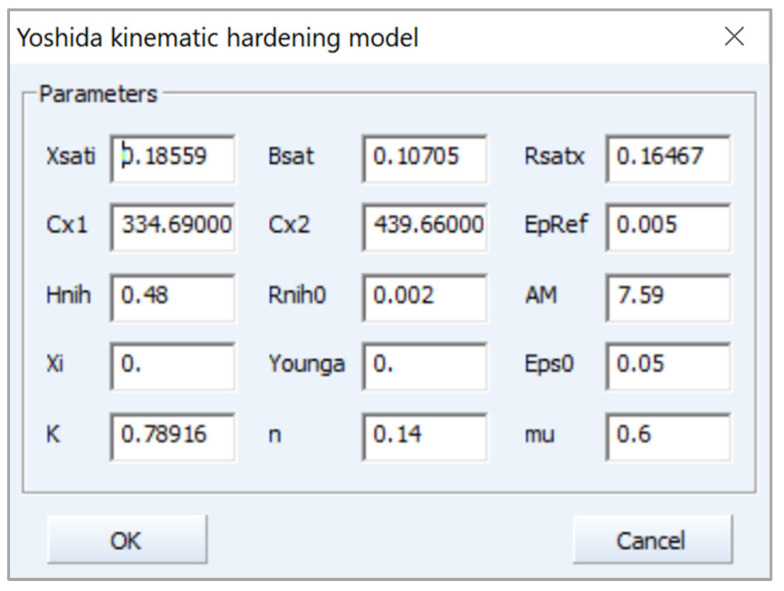
Definition of the Yoshida kinematic hardening model in the PS2G environment.

**Figure 26:**
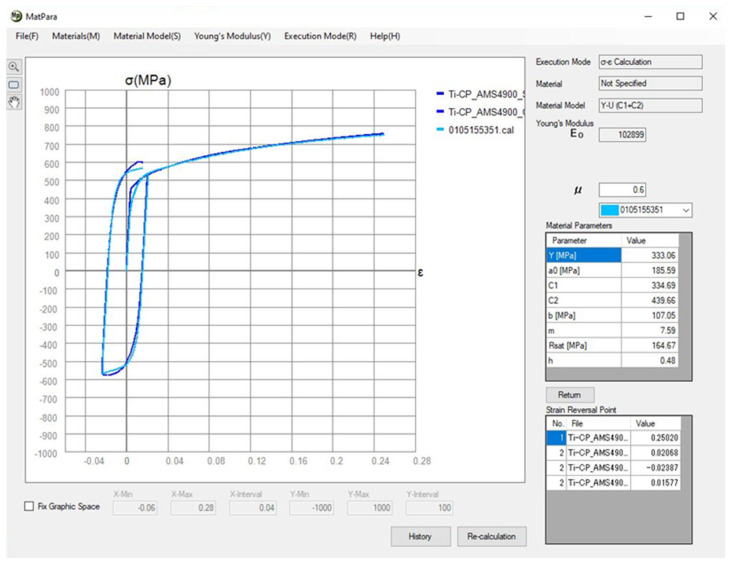
Example of fitting constants for Yoshida model in software MatPara.

In Figure 25 below, the Yoshida kinematic hardening model in the plastic deformation region is defined. This model is defined through several parameters and constants obtained using the external licenced software MatPara v2.1.0.0 in collaboration with MECAS ESI GROUP. In this case, the measured stress–strain curves from the static tensile test and cyclic test (the average dependence is obtained from five measurements) are interleaved with the approximation curve from the software and then the individual parameters and constants are determined by fitting (generating)—see Figure 26.

#### 3.3.2. Numerical Simulation of the Sheet Metal Process in PAM STAMP 2G Software

Using the numerical simulation of the sheet metal forming in PAM STAMP 2G software, selected forming processes corresponding to the real experiment on the stampings were simulated (see above). The process of producing U-shaped stamping was simulated first and then the process of drawing the strip of sheet over the draw-bead was conducted.

**Figure 27:**
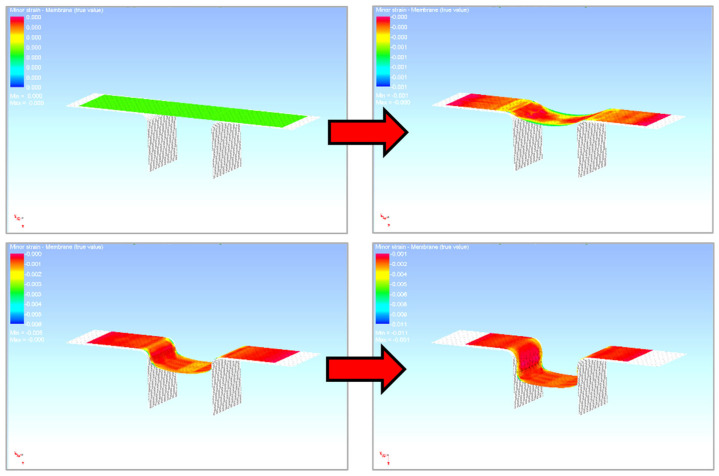
The bending process in the numerical simulation (software PAM STAMP 2G).

**Figure 28:**
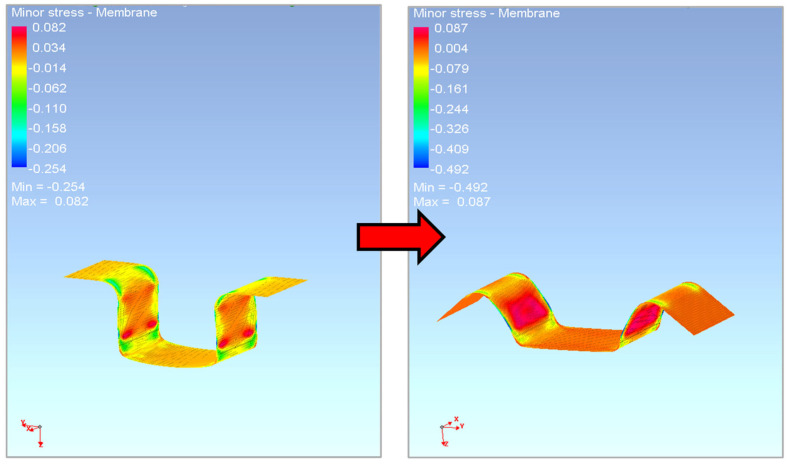
Numerical simulation of the stamping spring-back in the software PAM STAMP 2G.

So, the first simulated process was the forming of the sheet into a U shape. First, the functional parts of the tool and the blank were imported into the numeric simulation environment. These individual parts had to be fitted with a finite element mesh, their positions were determined and the kinematic quantities were entered for the calculation. The process and technological parameters of the numerical simulation were adjusted with respect to the parameters corresponding to the real experiment. First, the U-shaped bending process was simulated, and when the tool was released, the process of the subsequent material spring-back was computed—see Figure 27 and Figure 28.

As for the second simulated process, the drawing of the sheet strip over the draw-bead was conducted. For the implementation of the numerical simulation, it was necessary (as in the first case) to import the individual functional parts of the tool and the strip of sheet into the environment of the simulation software PAM STAMP 2G to create a finite element mesh to assign the positions, kinematic variables and process boundary conditions. Subsequently, the simulation was carried out according to the selected material computational model.

**Figure 29:**
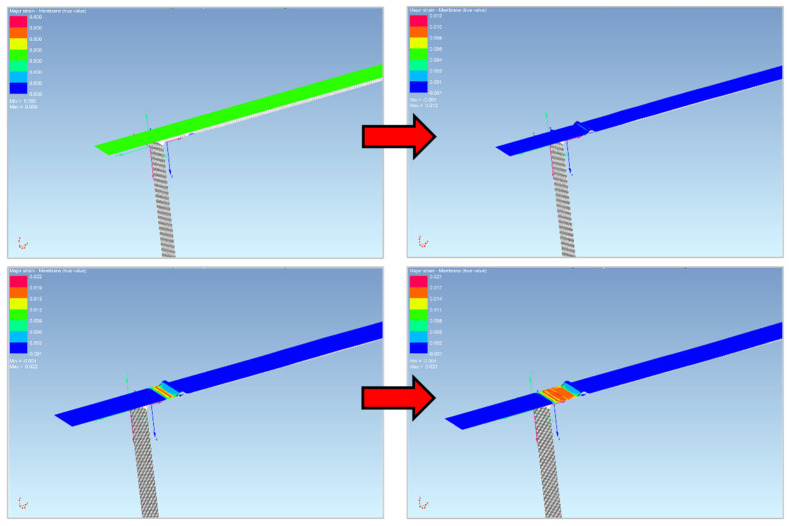
The bending process in the numerical simulation (software PAM STAMP 2G).

**Figure 30:**
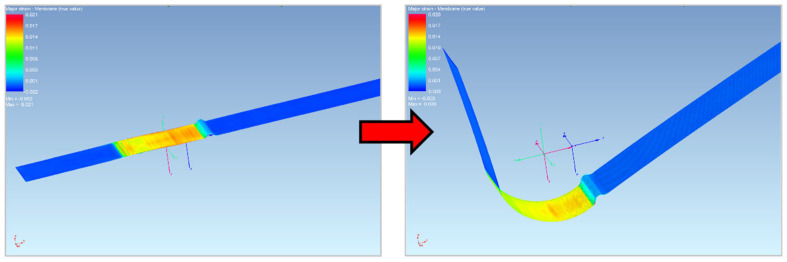
Numerical simulation of the stamping spring-back in the software PAM STAMP 2G.

Figure 29 and Figure 30 show the course of the numerical simulation of drawing the strip and also drawing the strip over the draw-bead after the subsequent material spring-back.

## 4. Discussion

In the following chapters, the results and a comparison of the numerical simulations with the real stamping process are shown and discussed. Furthermore, an evaluation of the selected computational models and numerical simulation parameters influence the forming process, and the subsequent spring-back of the tested Titanium AMS 4900 casting is presented.

**Figure 31:**
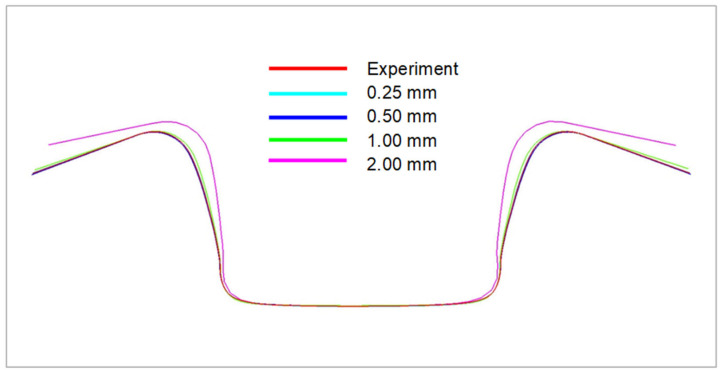
Comparison of deformation and subsequent spring-back for different mesh element sizes.

The basic parameter for the FEM calculation of numerical simulation is the appropriate choice of finite element size or element size and the settings of their meshing. It is generally known that the element size affects the accuracy of the numerical simulation quite significantly, i.e., a finer element mesh guarantees more accurate results and deformation and subsequent material spring-back. However, as the element size decreases, the computational time and hardware complexity of the entire computation also increases significantly. In view of these facts, it is advisable to choose a specific compromise between the used element size with respect to the computational complexity and the accuracy of the simulation results. The size of the elements should describe the shape of the forming tools with sufficient accuracy on the one hand and describe the deformation and spring-back results of the formed material with sufficient accuracy on the other hand. For this reason, a simple test on the influence of element size on the resulting accuracy of the simulation computation was carried out in comparison with already known sheet metal stamping. Figure 31 illustrates this comparison, where it can be observed that the element size does indeed have a significant effect on the final results. Based on this test, a mesh size equal to 0.5 mm in element dimension is chosen for further calculations, which sufficiently accurately describes the shape of the forming tools while maintaining high accuracy in the calculation of material deformation and subsequent spring-back. Using a finer mesh than 0.5 mm no longer provides any significant benefits in this case. With the need to speed up the calculation, it is possible to use a 1 mm mesh for common forming issues with sufficient accuracy.

**Figure 32:**
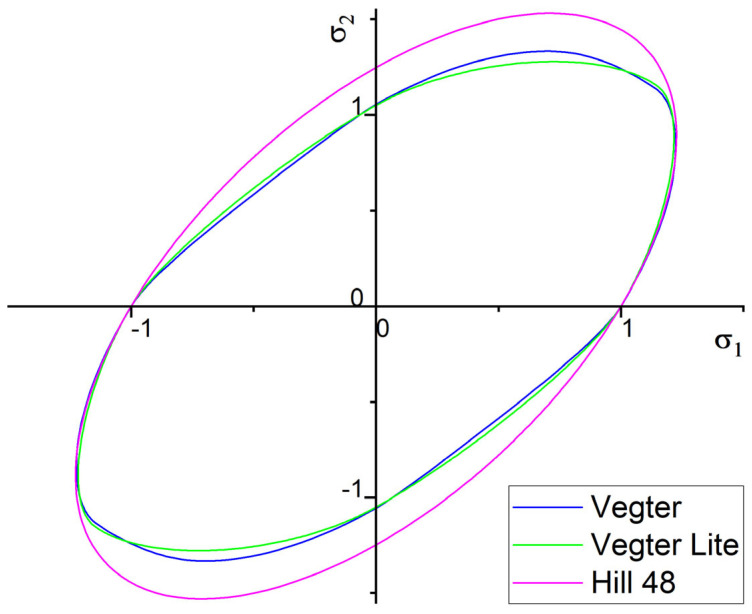
Comparison of selected material models representing plasticity condition for Ti-CP material AMS4900.

Another important parameter that affects the accuracy of the numerical simulation computation is the chosen material computational model. Figure 32 shows a comparison between the yield criteria boundaries in the plane of principal stresses σ_1_ and σ_2_ of the tested Vegter yield criterion and the simple Hill 48 yield criterion. This boundary of the relevant yield criterion is controlled through the selected material tests under the defined loading method as described above. It is possible to observe the effect of the material tests on this boundary, and it can also be observed that the more complex Vegter model has significant deviations from the simple Hill 48 yield criterion.

**Figure 33:**
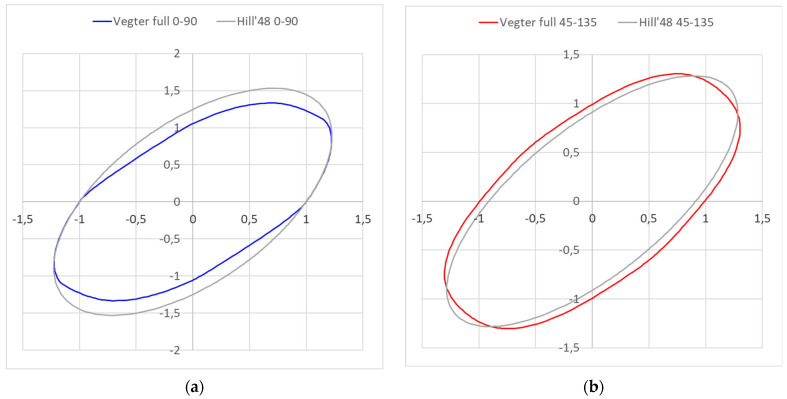
A comparison of the plasticity boundaries for Vegter Standard model and Hill 48 in the direction 0–90° (**a**) and 45–135° (**b**).

Figure 33 shows a comparison of the Vegter standard and Hill 48 yield criterion for the 0–90° (left) and 45–135° (right) directions. It can be seen here that in the 0–90° direction, the Vegter yield criterion is stricter compared to Hill 48 for the elastic–plastic transition. For example, in the 90° direction, Hill 48 transforms into the plastic state about 18% later than Vegter yield criterion, about 4% later for the biaxial loading and about 15% later for the pure shear loading. In contrast, the Hill 48 yield criterion is stricter in the 45–135° direction. The Hill 48 yield criterion is about 9% later in the 45° direction and about 11.5% later in the case of pure shear loading. In the case of biaxial loading, the Vegter model is stricter by approx. 4.5% in the 45–135° direction.

**Figure 34:**
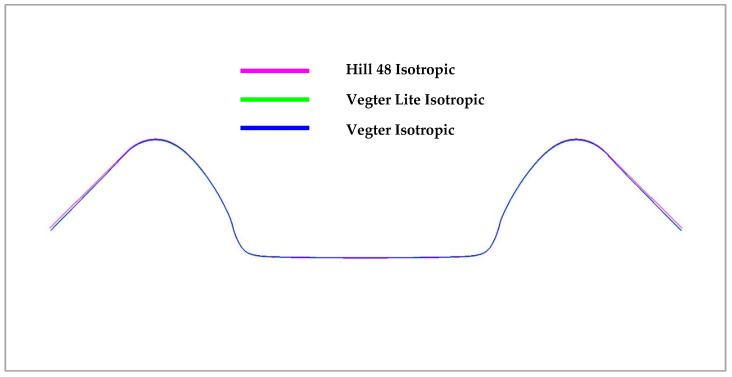
A comparison of the resulting contour obtained by the numerical simulation of Hill48, Vegter Lite and Vegter Standard with the isotropic hardening model for Ti-CP AMS4900.

With regard to the results above, the following figures illustrate the comparison of the numerical simulation of the forming process according to the selected material models and the subsequent comparison with the results of the real forming process—see the experiment. In the case of the computation for the sheet metal bending process into a U shape, Figure 34 shows the comparison of the resulting stamping contour obtained through numerical simulation in the software PAM STAMP 2G for every used material hardening model. It shows a comparison of the yield criterion Hill48 with the isotropic hardening model (magenta), Vegter Lite with the isotropic hardening model (green) and Vegter Standard also with the isotropic hardening model (blue). In this comparison, virtually no variation can be observed between the used yield criteria.

**Figure 35:**
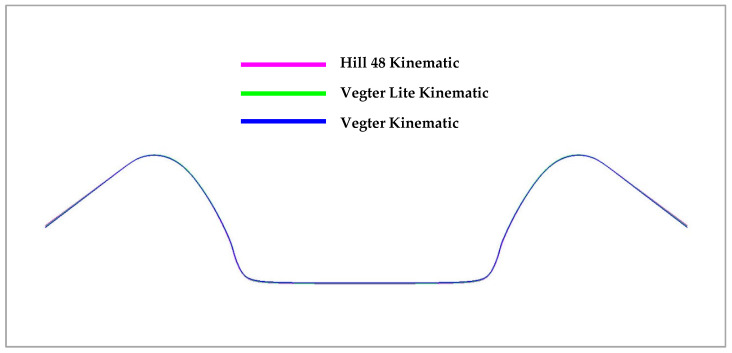
A comparison of the resulting contour obtained by the numerical simulation of Hill48, Vegter Lite and Vegter with the kinematic hardening model for Ti-CP AMS4900.

Figure 35 shows a comparison of the sheet contours obtained by numerical simulation in the software PAM STAMP 2G for Hill48 with the kinematic hardening model (magenta), Vegter Lite with the kinematic hardening model (green) and Vegter Standard with the kinematic hardening model (blue). In this comparison, almost comparable results are again seen with only a slight variation in the bottom part of the stamping.

**Figure 36:**
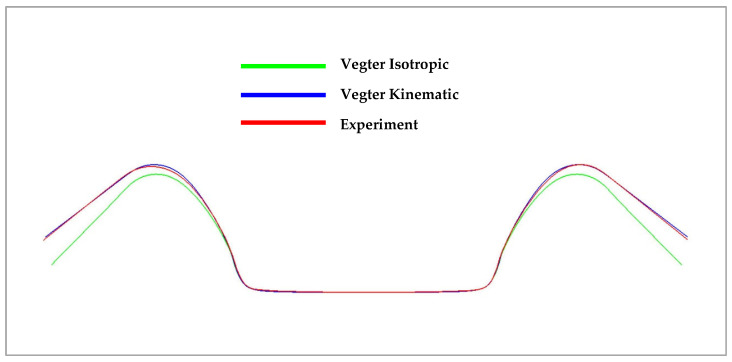
Comparison of resulting contour obtained by numerical simulation of Vegter Isotropic, Vegter Kinematic hardening model and real stamping for Ti-CP AMS4900.

In the figures above, it can be observed that in the case of a simple U-shaped bending process, where the deformation of the material is not too large or complex, the choice of the material model with regard to the relevant yield criterion does not have a significant influence on the resulting accuracy of material spring-back. Figure 36 below compares the isotropic (green) and kinematic (blue) hardening models during deformation and the contour obtained by real experiment (red). Here, it is already possible to observe the relatively significant deviations in the calculation of the spring-back between the individual computational models with respect to its comparison with the real contour of the stamping, where the variant with the kinematic hardening model during deformation describes much more accurately the shape and angle (and thus the spring-back) of the real stamping.

**Figure 37:**
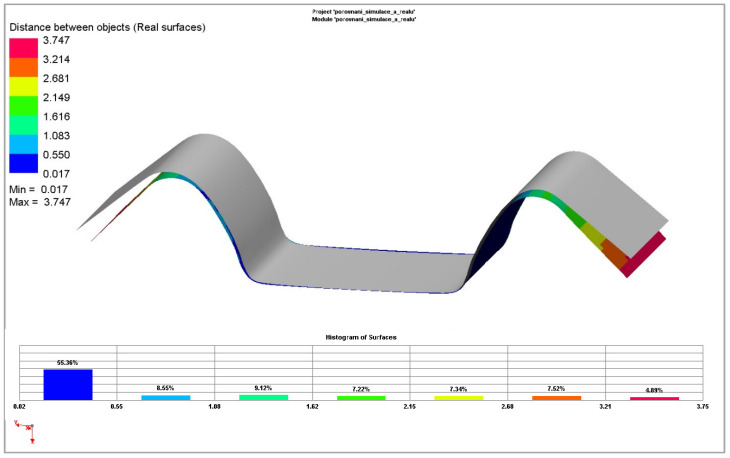
Comparison of contour deviations obtained by numerical simulation of Hill48 Isotropic hardening model and real stamping for Ti-CP AMS4900 material.

**Figure 38:**
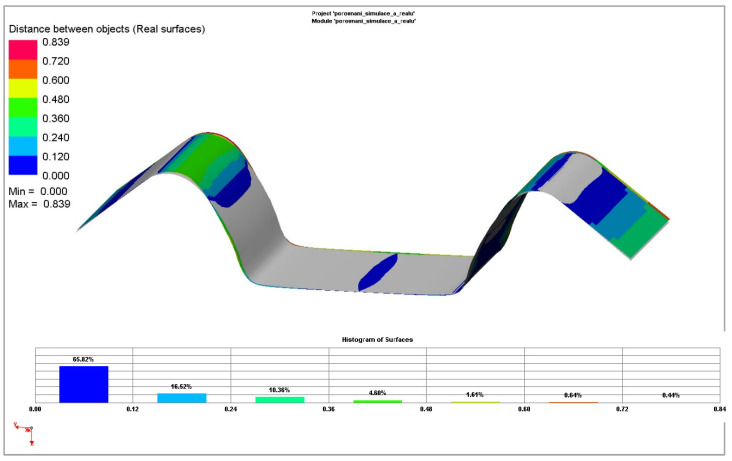
Comparison of contour deviations obtained by numerical simulation of Vegter Kinematic hardening model and real stamping for Ti-CP AMS4900 material.

Figure 37 and Figure 38 illustrate the deviations obtained compared to the real contour of the experimental stamping. The deviations of the simplest yield criterion Hill 48 combined with the isotropic hardening model are shown, as well as deviations of the advanced and most accurate yield criterion Vegter Standard combined with the kinematic hardening model during plastic deformation.

In the second simulated process of drawing a strip of sheet over the draw-bead, it is already possible to observe quite noticeable differences between the individual models of the numerical simulation and the contour of the real stamping obtained by the experiment. This result corresponds with the established assumption that, in this process, the material undergoes much larger and complex deformation when passing over the draw-bead, i.e., the Bauschinger effect should be significantly taken into account, and thus, the influence of the differences between the individual computation models should become evident.

**Figure 39:**
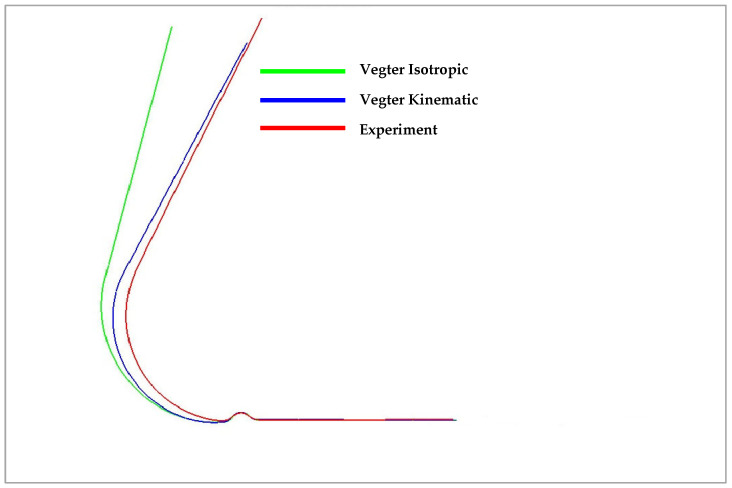
A comparison of the resulting contour of the stamping obtained by the numerical simulation of Vegter Lite and Vegter Standard with the kinematic hardening model and the contour of the real stamping for Ti-CP AMS4900.

Moreover, in the case of testing the Ti-CP AMS4900 titanium alloy, the isotropic hardening models completely fail in the spring-back calculation of this problem. Only the kinematic hardening model (see Figure 39) was able to cope with this task, complete the computation and thus describe the course of material spring-back. But this was only in combination with the yield criteria, Vegter Standard and Vegter Lite, which again highlights the advantages of using a kinematic hardening model during deformation in combination with the advanced yield criterion of Vegter. The Hill 48 model was unable to cope with this task in any used variant, as indicated by the very different boundary of the Hill 48 yield criterion boundary compared to the Vegter models, as can be seen in Figure 32 and Figure 33.

In Figure 39, it is possible to observe the results of the numerical simulation for the drawing of a sheet strip over the draw-bead for the selected material models with the contour of a real titanium alloy stamping obtained from the experiment. Specifically, the Vegter (blue) and Vegter Lite (green) yield criteria with the kinematic hardening model variant in the plastic deformation region and the contour of the real stamping (red) are compared. It can be observed that the most complex yield criterion (Vegter Standard) in the variant with the kinematic hardening model (the so-called Yoshida model) comes closest to the real result despite the truly extreme spring-back of the tested titanium alloy.

Numerical simulation using the finite element method always introduces a certain degree of inaccuracy with respect to the real technological process. The reason for this inaccuracy is mainly due to the fact that the standard continuous environment inside the formed material in the real forming process is replaced by means of mesh elements of a chosen size when calculating the numerical simulation of the given process. This fact affects the accuracy of the numerical simulation quite considerably. Other possible errors, and the degree of inaccuracy introduced into the numerical calculation, are influenced by the form of the chosen material computational model, which is described above. In terms of the choice of the relevant computational model, it can be seen that the Vegter model outperforms the simple model Hill 48. The reason for the refinement of the results is that the plasticity boundary for the Vegter model is defined by multiple material tests, taking into account the different states of stress in the deformed material compared to the Hill model, which takes into account only the results obtained by static tensile testing. This difference in these yield criteria is shown in Figure 32 and Figure 33. The difference in the elastic to plastic transition obviously also affects the resulting material’s spring-back. For industrial practice, this fact means that it is advisable to choose more complex material tests for the numerical simulation of materials, revealing the high magnitude of spring-back to more accurately define the boundary of the relevant yield criterion in advanced material models. Another influencing factor for the accuracy of the numerical calculation may be the fact that in the real forming process, the elastic modulus *E* changes during the repeated change in the load direction during the deformation of the material; similarly, the value of the constant friction coefficient may not be observed in the real forming process. The combination of all these and other aspects gives the overall distortion level of numerical simulations when compared with the real sheet metal forming process.

## 5. Conclusions

The research published in this paper, in the experimental part, focused primarily on the deformation analysis and description of the deformation behaviour of Titanium AMS4900 under different loading conditions and defined stress states in the tested material. The results of this testing are used to properly define the yield criteria of the material and to describe the material behaviour in terms of primary and developed plastic deformations. The output material characteristics and selected properties were further applied to define and correctly set up the material computational models used in the numerical simulation. When we focused on the numerical simulation of the sheet metal forming process, two simple forming processes were tested here. The first process of bending the metal strip into a U shape represented a simple deformation process, where the strain and complex deformation were not too large. Then, the process of drawing a strip of sheet metal over a draw-bead occurred, where the material underwent rather significant deformation and multiple changes in state when passing over the draw-bead. These simulation processes of deformation and the subsequent material spring-back were carried out in the software PAM STAMP 2G with respect to the selected material computational models and with respect to the conditions of producing the real stamping, which were used for the comparison and evaluation of the numerical simulation results.

From the results of experimental part and subsequently performed numerical simulations, it is possible to make conclusions that the dominant influencing factor for the accuracy of the numerical simulation is certainly the choice of the material computational model. This fact influences both the course of the deformation computation itself and also the correct prediction of the subsequent material spring-back after the forming process. Of the tested numerical simulation yield criteria, the Vegter Standard yield criterion proves to be the most accurate. The advantages of the Vegter model are evident both in the determination of the yield criterion boundary condition and consequently in the area of primary and developed plastic deformations. From the results discussed, it is clear that the Vegter yield criterion in combination with a kinematic hardening model should be chosen for the most accurate prediction of the spring-back in these cases.

The determined and verified results of this experimental research and numerical simulations can generally contribute to the refinement of the results from numerical simulation processes in the real practice of forming sheet metal stampings. From a practical application point of view, these results are relevant in the field of materials or new types of materials used in practice and also for possible types of solutions with regard to the shape of the produced sheet metal parts. The use of numerical simulation processes in production can also support the economic side of production or reduce the production times in the whole production chain. The results further demonstrate the potential applications of titanium alloys in the stamping process and industrial practice of the stamping process. The titanium forming process entails a lot of complications and this research can certainly contribute to the facilitation of this process in conjunction with the possibility of applying mathematical modelling to predict selected parameters and characteristics, thus facilitating the introduction of these special material types into industrial practice. In the field of scientific knowledge, these results reveal new aspects of the research of issues in the field of materials, material testing and numerical simulations of the forming process. Furthermore, these results can serve as a basis for further research in the field of material science and the mathematical modelling of sheet metal forming processes.

## Figures and Tables

**Table 1:** Basic mechanical properties of Titanium AMS 4900.

Rolling Direction (°)	*R*_p0.2_ (MPa)	*R*_m_ (MPa)	*A*_g_ (-)	*A*_80mm_ (-)	*E* (MPa)
0	464.0 ± 1.2	599.5 ± 1.1	0.1329 ± 0.0018	0.2513 ± 0.0021	102,899 ± 118
45	521.0 ± 1.0	577.5 ± 1.1	0.0973 ± 0.0021	0.2551 ± 0.0028	109,420 ± 124
90	573.0 ± 1.1	624.0 ± 1.0	0.1294 ± 0.0012	0.2530 ± 0.0019	116,215 ± 115

**Table 2:** Approximation constants determined by the Krupkowski approximation of the stress– strain curve from the static tensile test of Titanium AMS 4900.

Rolling Direction (°)	C (MPa)	*n* (-)	φ_0_ (-)	*R* (-)
0	948.7 ± 0.1	0.1636 ± 0.0001	0.01229 ± 0.00002	0.87 ± 0.01
45	887.1 ± 0.3	0.1482 ± 0.0002	0.01779 ± 0.00007	2.36 ± 0.01
90	1089.9 ± 0.6	0.2226 ± 0.0004	0.03746 ± 0.00014	2.61 ± 0.01

**Table 3:** Approximation constants determined by the Krupkowski approximation of the stress-strain curve from the HBT of Titanium AMS 4900.

Rolling Direction (°)	C (MPa)	*n* (-)	φ_0_ (-)	*R* (-)
-	1487.2 ± 10.0	0.2980 ± 0.0067	0.03030 ± 0.00273	1.15 ± 0.01

**Table 4:** Approximation constants determined by the Krupkowski approximation of the stress- strain curve from the plane strain tensile test of Titanium AMS 4900.

Rolling Direction (°)	C (MPa)	*n* (-)	φ_0_ (-)
0	1051.5 ± 6.4	0.1257 ± 0.0023	0.00671 ± 0.00043
45	1163.1 ± 7.1	0.1466 ± 0.0030	0.01773 ± 0.00090
90	1242.7 ± 16.8	0.1912 ± 0.0093	0.04621 ± 0.00415

**Table 5:** Approximation constants determined by the Krupkowski approximation of the stress-strain curve from the shear test of the titanium alloy AMS 4900.

Rolling Direction (°)	C (MPa)	*n* (-)	φ_0_ (-)
0	448.1 ± 0.6	0.1033 ± 0.0012	0.00093 ± 0.00098
45	509.0 ± 0.3	0.1668 ± 0.0014	0.02833 ± 0.00176
90	491.2 ± 0.2	0.5935 ± 0.0007	0.03008 ± 0.00086

**Table 6:** Yield strength at changing tensile–compression loadings.

Rolling Direction (°)	*R*_p0.2_ (MPa)	*R*^C^_p0.2_ (MPa)	Difference *R*_p0.2_ (%)
0°	464.0 ± 0.1	−304.9 ± 0.2	34.3 ± 0.1

## Data Availability

The original contributions presented in the study are included in the article, further inquiries can be directed to the corresponding author.
